# Theoretical and Experimental Study of 13.4 kV/55 A SiC PiN Diodes with an Improved Trade-Off between Blocking Voltage and Differential On-Resistance

**DOI:** 10.3390/ma12244186

**Published:** 2019-12-12

**Authors:** Yuewei Liu, Ruixia Yang, Yongwei Wang, Zhiguo Zhang, Xiaochuan Deng

**Affiliations:** 1 School of Electronics and Information Engineering, Hebei University of Technology, Tianjin 300401, China; 2 School of Electrical and Electronic Engineering, Shijiazhuang Tiedao University, Shijiazhuang 050043, China; 3 The 13th Research Institute, CETC, Shijiazhuang 050051, China; 4 School of Electronic Science and Engineering, University of Electronic Science and Technology of China, Chengdu 610054, China

**Keywords:** silicon carbide, field limiting ring, breakdown voltage, Baliga’s figure-of-merit

## Abstract

In this paper, a 13.4 kV/55 A 4H-silicon carbide (SiC) PiN diode with a better trade-off between blocking voltage, differential on-resistance, and technological process complexity has been successfully developed. A multiple zone gradient modulation field limiting ring (MGM-FLR) for extremely high-power handling applications was applied and investigated. The reverse blocking voltage of 13.4 kV, close to 95% of the theoretical value of parallel plane breakdown voltage, was obtained at a leakage current of 10 μA for a 100 μm thick, lightly doped, 5 × 10^14^ cm^−3^ n-type SiC epitaxial layer. Meanwhile, a fairly low differential on-resistance of 2.5 mΩ·cm^2^ at 55 A forward current (4.1 mΩ·cm^2^ at a current density of 100 A/cm^2^) was calculated for the fabricated SiC PiN with 0.1 cm^2^ active area. The highest Baliga’s figure-of-merit (BFOM) of 72 GW/cm^2^ was obtained for the fabricated SiC PiN diode. Additionally, the dependence of the breakdown voltage on transition region width, number of rings in each zone, as well as the junction-to-ring spacing of SiC PiN diodes is also discussed. Our findings indicate that this proposed device structure is one potential candidate for an ultra-high voltage power system, and it represents an option to maximize power density and reduce system complexity.

## 1. Introduction

There is no doubt that 4H-silicon carbide (SiC) material will play an increasingly important role in ultra-high voltage supply system or low-power loss industrial applications, given its excellent intrinsic structure and physical properties, especially in the future smart grids which contain high voltage DC power distribution and flexible AC transmission [1,2,3]. Research has reported that 4H-SiC power devices have excellent performance in both 50–150 kW DC power supplies and 60–126 kV voltage supplies [4,5,6]. Compared with their silicon counterparts, SiC power transistors and diodes could decrease the series connection of multiple Si devices, thus reducing the enormous power dissipation and extensive water cooling [7,8]. For example, a typical voltage of power distribution is 6.5–7.2 kV, where 13–15 kV power transistors and diodes are required for constructing single-phase converters [9]. It is widely known that the trade-off between specific on-resistance and breakdown voltage is a key issue related to the design and fabrication of an ultra-high voltage device. Conductivity modulation effects make PiN bipolar devices have higher conductance characteristics, which shows that PiN rectifiers have lower on-resistance than 10 kV-class unipolar SiC diodes (>100 mΩ·cm^2^) in many intensive studies [10,11]. In these studies, the multi-zone junction termination extension (JTE) technique—involving space-modulated JTE, multiple ring modulated JTE, mesa-etched JTE, and Hybrid JTE—is the typical edge termination technology for achieving a high blocking efficiency [12,13,14,15]. However, JTE structure suffers from a narrow optimum implantation dose window and SiC surface charge, which leads to breakdown voltage degradation and reliability problems. The simple preparation technology of the field limiting ring, which is fabricated simultaneously with the main junction, reduces manufacturing costs. Fabrication benefits could lead to the field limiting ring becoming one of the most popular and most commonly used terminal structures, compared to the JTE edge terminal that requires multiple injections. Unfortunately, field limiting rings are often difficult to optimize and fabricate for ultra-high voltage SiC devices, because of their simple structure. Up to now, reports on SiC PiN diodes with extremely high-power capabilities (both ultra-high voltage and high forward current) are still limited [16,17].

In this paper, we proposed and demonstrated a large area SiC PiN diode rated at 10 kV using a novel multi-zone gradient modulation field limiting ring (MGM-FLR) structure without increasing the complexity of the technological process or the cost of the processing steps. The reverse blocking voltage of 13.4 kV, close to 95% of the theoretical value of parallel plane breakdown voltage, was obtained at a leakage current of 10 μA for a 100 μm thick lightly doped 5 × 10^14^ cm^–3^ n-type SiC epitaxial layer. Furthermore, a differential on-resistance at 10 A (100 A/cm^–2^) is only 4.1 mΩ·cm^2^ (2.5 mΩ·cm^2^ at 550 A/cm^2^) at room temperature. The highest Baliga’s figure-of-merit (BFOM) is achieved as high as 72 GW/cm^2^ for the fabricated SiC PiN diode.

## 2. Device Structure and Fabrication

**Figure 1:**
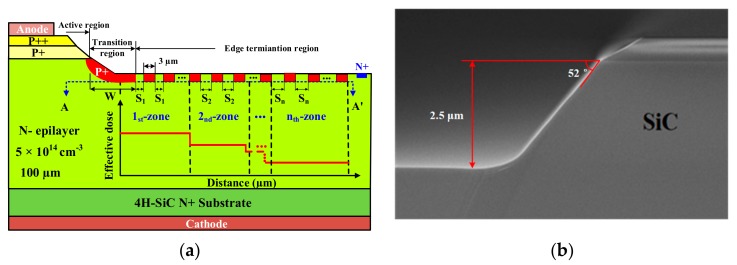
(**a**) Schematic cross-section of a 4H-SiC PiN diode with multiple zone gradient modulated field limiting ring (MGM-FLR); (**b**) mesa structure.

In this work, an N-drift region thickness of 100 μm and doping concentration of 5 × 10^14^ cm^–3^ was designed to achieve a 10 kV-class blocking voltage. In addition, the PiN structure also contains a P^++^ contact epi-layer (>10^19^ cm^–3^, 0.5 μm), a P^+^ epi-layer (2 × 10^18^ cm^–3^, 1 μm) and a low-resistivity N^+^ substrate. Figure 1a illustrates a simplified cross-section view of the proposed 4H-SiC PiN rectifier with mesa combined with MGM-FLR structure. The mesa structure of 2.5 μm height and a 52° angle was formed by ICP (inductive coupled plasma) etching with CF_4_-O_2_ Ar gas, where a hard mask of SiO_2_ was used. An ICP coil power of 750 W and a bias platen power of 150 W were employed to form the gradual surface. The etch rate and selectivity were typically 280 nm/min and 1.8:1, respectively. From Figure 1b, a rounded corner at the mesa bottom can be seen.

**Figure 2:**
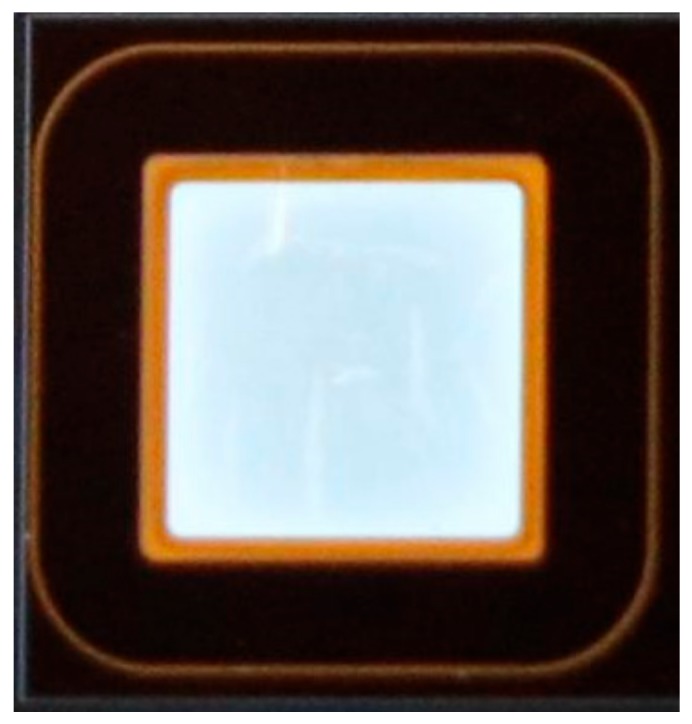
Optical microscopic image of the fabricated SiC PiN diode with an active area of 0.1 cm^2^ (0.28 cm^2^ chip size).

The multi-step Al ion injection of the edge terminal multi-zone field limiting ring, which mitigates the main junction electric field concentration effect, was performed at a temperature of 500 °C and a maximum implantation energy of 500 keV. The implantation concentration and depth of P-rings implantation were 2 × 10^19^ cm^–3^ and 0.6 μm, respectively. After several simulations with the 2-D Silvaco-TCAD tool (V. 4.4.3.R, Silvaco, Santa Clara, CA, USA), the optimal structure was confirmed as 12 × 11 rings, which means 12 regions and 11 rings per region. The space of each zone increases by a coefficient of 0.2 μm with the fixed ring width of 3 μm, on the basis of S_1_ = 1.3 μm (S_1_ is the gap of P^+^-type implantation rings in the first zone). Therefore, the total length of the edge termination region is 715 μm. For the fabrication of SiC power devices, an important and difficult process was high-temperature annealing, compared to conventional Si device processes. After the injection of the field limit ring, which formed simultaneously with the main junction, the 1700 °C, 30 min anneal with a carbon cap was carried out in an Ar atmosphere to active the implanted ions. Subsequently, there were two passivation layers deposited. The material of the first layer was 500 Å thermally grown oxide and a 1 μm-thick TEOS oxide film by LPCVD (low pressure chemical vapor deposition). The second layer was composed of polyimide layers deposited after the formation of anode and cathode ohmic electrodes, which were Ti/Al and Ni annealed at 1000 °C for 2 min. Figure 2 shows the optical microscopic image of the fabricated SiC PiN diode with an active area of 0.1 cm^2^ (0.28 cm^2^ chip size). In this paper, device characteristic analysis was performed by the Atlas from Silvaco-TCAD [18]. The SiC physical models, such as impact ionization, band gap narrowing, incomplete ionization of impurities, mobility, and generation–recombination, were adopted based on the latest literature on SiC.

## 3. Experimental Results and Discussion

**Figure 3:**
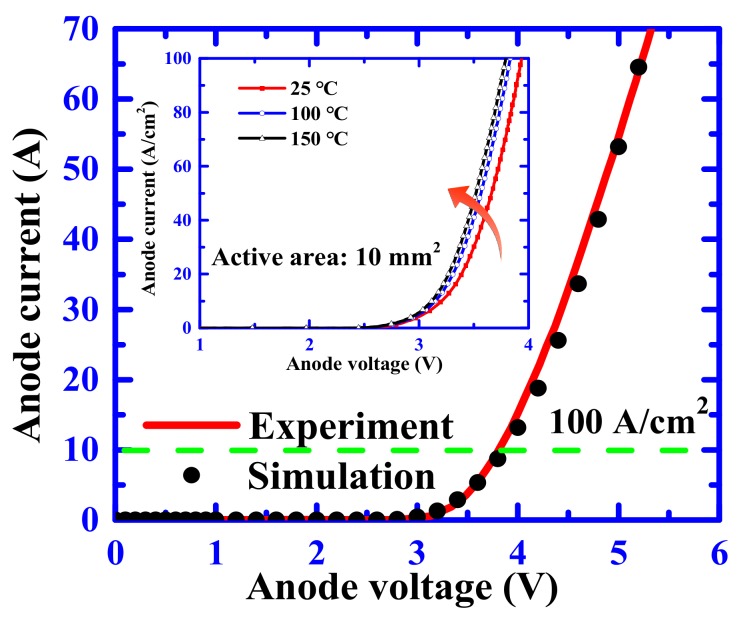
I–V characteristics of the proposed 4H-SiC PiN diode different temperature. Solid lines are experimental curves and solid markers are simulation results.

Figure 3 plots the wafer-level forward output characteristics for the fabricated SiC PiN diode using the Keysight B1505 curve tracer (Agilent, Palo Alto, CA, USA) with the maximum current limit of 70 A. It was found that the forward current of a SiC device with a chip size of 0.28 cm^2^ (active area of 0.1 cm^2^) was 55 A, biased at a 5 V forward voltage, which showed high conductivity characteristics for the fabricated SiC PiN diode. The differential on-resistance value of the 4H-SiC PiN device with active area of 0.1 cm^2^ can be calculated as 2.5 mΩ∙cm^2^ at a current of 55 A (4.1 mΩ∙cm^2^ at a current density of 100 A/cm^2^).

The dependence of the forward current on the temperature from 25 °C to 150 °C is illustrated in the inset of Figure 2. The forward voltage at a temperature of 25 °C was 3.8 V, while the forward voltage at 150 °C was 3.7 V at a current density of 100 A/cm^2^. The slight increase in forward current demonstrated that the fabricated device exhibits outstanding forward performance with temperature changes. We concluded that the forward voltage dropped with the increase of temperature because the SiC band gap, narrowing with the higher temperature, caused the reduction of the p–n built-in voltage and the increase of carrier lifetimes with temperature. From Figure 3, it is also clear that the experimental values represented had the same trend and similar values compared to the simulation results. Therefore, the minority carrier lifetime was estimated to be 2.4 μs for the as-grown SiC epitaxial material.

**Figure 4:**
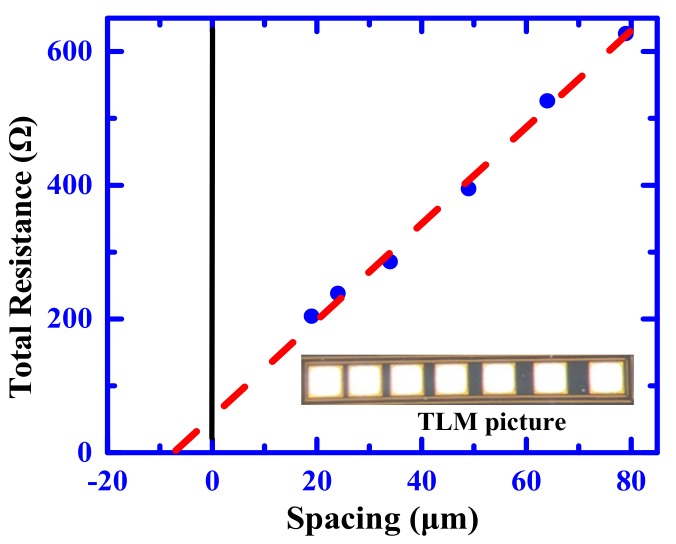
Measured resistance plotted against the contact spacing (linear transfer length method (TLM)) for Ti/Al/P-type 4H-SiC.

The relatively inferior point of the ultra-high voltage SiC PiN diode is that it has a high forward voltage drop due to its material characteristics. In addition, it was difficult to form a lower contact resistivity to P-type SiC than that of N-type SiC. This is because an ideal contact metal with a work function to N/P-type SiC is about 4 eV and 7 eV, respectively [19]. I–V characteristics measured against the contact spacing at different positions on the wafer are plotted in Figure 4. It was found that a contact resistivity for P^+^-type SiC, down to about 10^–5^ Ω∙cm^2^, could be obtained with the linear transfer length method (TLM), which indicated a relatively good ohmic contact in comparison with the value of P-type contact resistivity reported.

**Figure 5:**
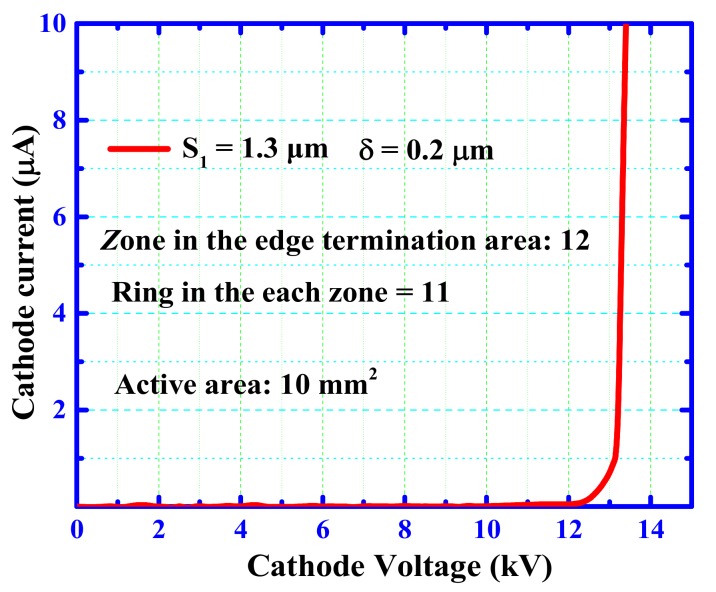
Measured blocking voltage performance of the fabricated SiC PiN rectifier.

Figure 5 shows the results of the reverse blocking performance test of the typical fabricated 4H-SiC PiN diode with MGM-FLR structure, which applied an ultra-high voltage measurement system that included an Agilent B1505 Curve Tracer (Agilent, Palo Alto, CA, USA), a Glassman FC20P6 high-power supply (XP Power, Singapore), and a Cascade Microtech probe (Cascade Microtech, Beaverton, OR, USA). The test data of leakage current below 10 kV came from the Agilent B1505, and the value of the leakage current beyond 10 kV came from the Glassman FC20P6 high-power supply, because the breakdown voltage could only be measured up to 10 kV due to limitations of the Agilent B1505. The test chips were dipped in Fluorinert oil to avoid the device discharging in the air. The maximum breakdown voltage measured at an anode leakage current of 10 μA was as high as 13.4 kV. It should be noted that the reverse blocking capabilities of the proposed device are approximately 95% of the ideal parallel plane junction, which can be calculated by Konstantinov’s formula [20,21]. A relatively low reverse leakage current density of 0.1 mA/cm^2^ indicated that the chip has excellent device blocking characteristics. Additionally, the fabricated SiC PiN diode exhibited a significantly higher Baliga’s figure-of-merit (BFOM, BV^2^/R_on,sp_) of 72 GW/cm^2^ [22].

**Figure 6:**
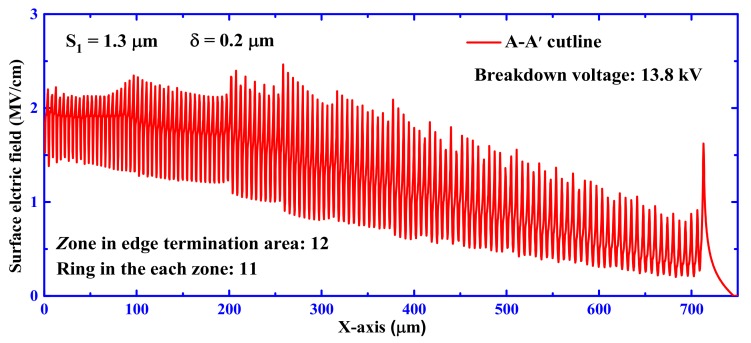
Simulated surface electric field distribution at off-state breakdown for a SiC PiN diode with MGM-FLR structure.

In order to understand the origin of the proposed devices with a near-theoretical breakdown voltage, the simulated 2-D electric-field profile is shown in Figure 6. The starting point of the A-A’ cutline in the figure is the position of the edge termination structure, at a distance of about 10 Å from the P^+^ region. The proposed multi-zone terminal field-limited ring structure (especially at S_1_ = 1.3 μm) makes the electric field show a trend of uniform reduction, which alleviates the electric field concentration effect. This structure prevents the device from breaking down at the main junction in advance and thereby increases the reverse blocking voltage characteristics of the device. It can be seen from Figure 5 that the highest electric field value of 2.5 MV/cm appears around 250 μm, which is the middle part of the terminal.

**Figure 7:**
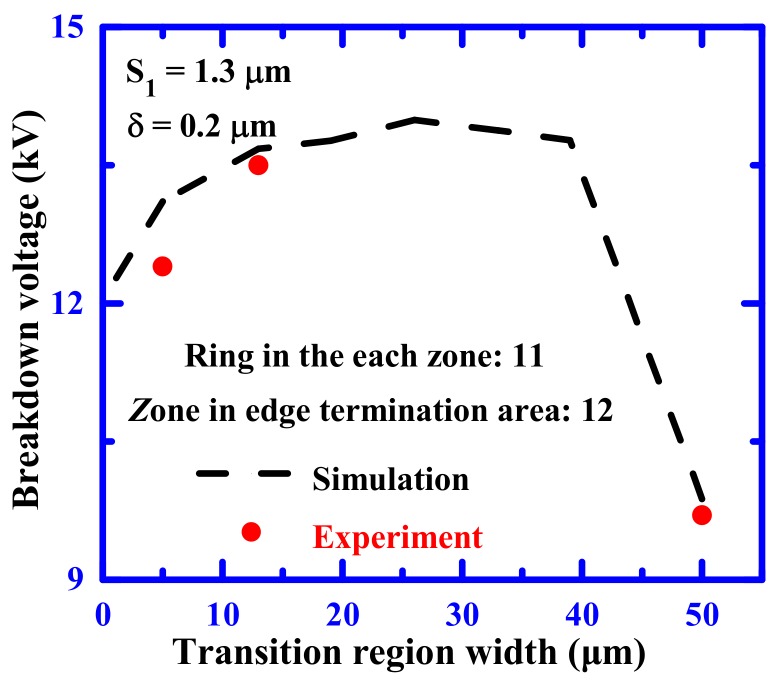
Transition region width influence on reverse blocking characteristics of the SiC PiN diodes.

Figure 7 illustrates the influence of transition region width on reverse blocking voltage of the MGM-FLR 4H-SiC PiN diodes. The dotted line shows the simulated results and the markers are the experimental results of the fabricated 10 kV-class SiC PiN diodes. The value of breakdown voltage increases at first, due to the increasing width of the transition zone, and then becomes less sensitive to the transition region width. However, the value of breakdown voltage sharply decreases when the transition zone width is larger than 40 μm. This is because the peak value of the electric field at the main junction could not be effectively shielded by the two high values of the transition region width. Obviously, the experimental values represented have the same trend and similar values compared to the simulation results.

**Figure 8:**
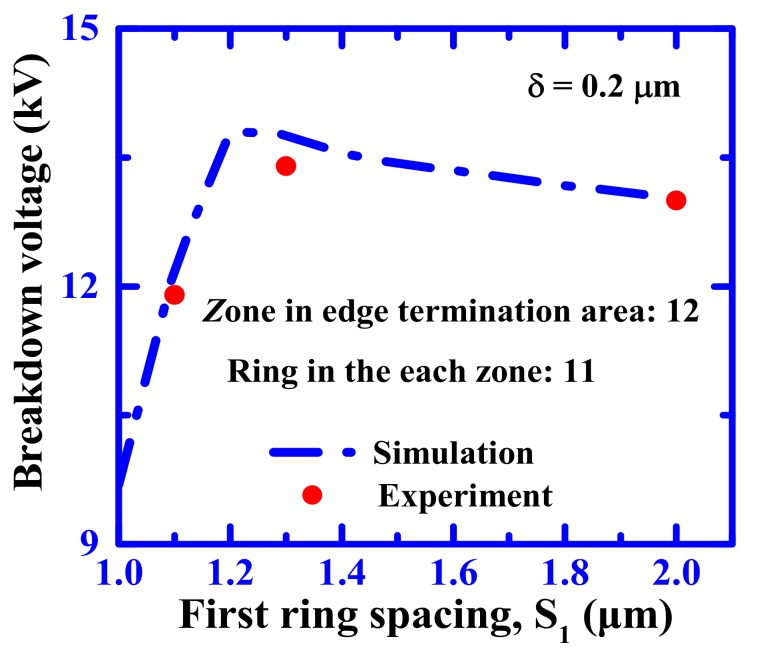
Dependence of the breakdown voltage on the junction-to-ring spacing of the SiC PiN diode.

It is generally known that the gap between the floating limited rings plays a significant role with regard to the reverse blocking characteristics for the SiC power device, because of a low junction depth of floating limited rings. Figure 8 plots the dependence of the breakdown voltage on the junction-to-ring spacing (S_1_) for a SiC PiN diode with MGM-FLR structure. The dotted line shows the simulated results, and the markers show the experimental results of the fabricated 10 kV-class SiC PiN diodes. The total length of the edge termination region increased with the increase of ring spacing. It is clear that the optimum breakdown voltage depends on the first ring’s spacing. The breakdown voltage reached the maximum when S_1_ = 1.2 μm, but slightly decreased as the junction-to-ring spacing decreased. For the fabricated SiC PiN diodes, the optimized junction-to-ring spacing was set to 1.3 μm, which is larger than the simulation value due to a possible photolithographic alignment error. It was also found that the experimental value represented had the same trend and a similar value compared to the simulation results.

**Figure 9:**
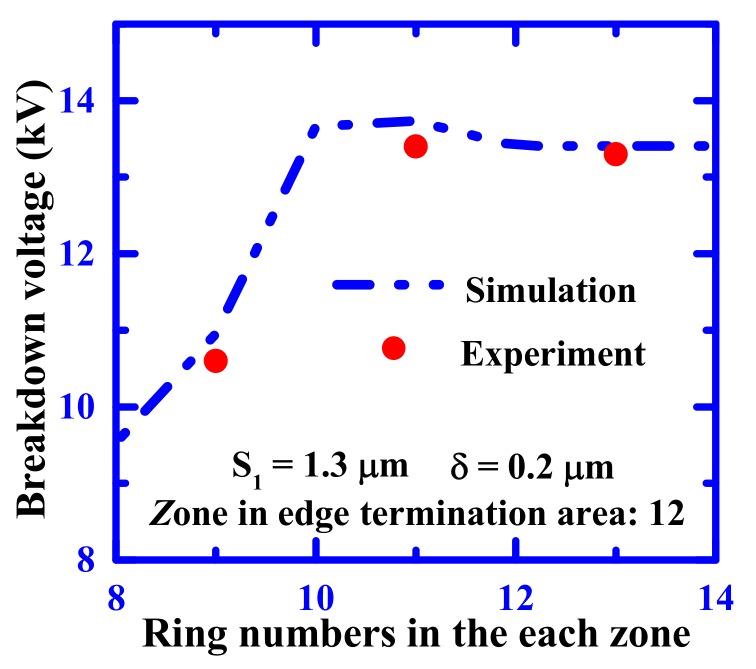
Relationship between the blocking voltage and the various number of rings in each zone.

Figure 9 shows the relationship between the number of rings in each zone and the blocking voltage of the SiC PiN diode. The total length of the edge termination region increased with the increase of p-ring numbers. As can be observed in the figure, the blocking voltage of the fabricated devices with a fixed S_1_ = 1.3 μm, δ = 0.2 μm, and 12 zone increased gradually when the number of rings in each region increased. The value reached a saturation value for 10 rings. It was also found that the experimental value represented had the same trend and a similar value to the simulation results.

In order to evaluate the proposed device characteristics, the performance comparison for the latest literature on the fabricated 4H-SiC PiN diodes is summarized in Table 1. It should be pointed out that the proposed SiC PiN diodes showed a better trade-off with regard to blocking voltage, differential on-resistance, and technological process complexity. In addition, the fabricated SiC device exhibited a significantly higher Baliga’s figure-of-merit than that reported by the SiC PiN diodes.

## 4. Conclusions

In this work, we proposed a novel edge terminal structure of multiple zone gradient modulated field limiting rings to make an electric field evenly distributed, to achieve > 10 kV-class 4H-SiC PiN diodes with mesa structures. The reverse blocking voltage of 13.4 kV at 10 μA could theoretically reach a value of up to 95% (calculated using the 100 μm drift thickness and a 5 × 10^14^ cm^–3^ concentration). At the same time, the forward voltage was 5 V and the forward current was 55 A, with the active area of 0.1 cm^2^. Differential on-resistance of 2.5 mΩ·cm^2^ and the highest Baliga’s figure-of-merit (BFOM) of 72 GW/cm^2^ was obtained for the fabricated 4H-SiC PiN device. Furthermore, the dependence of the breakdown voltage on transition region width, number of rings in each zone, as well as the junction-to-ring spacing of the SiC PiN diodes was also investigated. Our proposed device provides a simple and highly promising way to fabricate an ultra-high voltage (>10 kV) SiC power device.

## Figures and Tables

**Table 1:** Performance comparison of fabricated 10 kV-class SiC PiN diodes.

Reference	Current	BV (kV)	V_f_ at 100 A/cm^2^	Termination	R_on,sp_	BFOM BV^2^/R_on,sp_	Fabrication
			(V)	Efficiency	(mΩ·cm^2^)	(GW/cm^2^)	Complexity ^*^
[23]	>50 A	12.9	3.75	84%	5.75	29	Simple
[24]	~10 A	15	4.1	95%	25.5	9	Medium
[25]	<1 A	>10	3.3	-	3.4		Complex
[9]	<1 A	13	3.22	84%	1.87	90	Complex
[12]	<1 A	27.5	-	83%	-	-	Complex
This work	>50 A	13.4	3.8	95%	2.5	72	Simple

* Fabrication Complexity: “Simple” for one P-type implantation and without the process of enhancement of carrier lifetime, “Medium” for two P-type implantations and the process of enhancement of carrier lifetime, and “Complex” for multi-step implantation and the process of enhancement of carrier lifetime.
